# The long-term outcomes of local tumor destruction versus partial nephrectomy for cT1a non-clear cell renal cell carcinoma and development of prognostic nomograms

**DOI:** 10.1007/s00432-023-05571-8

**Published:** 2024-03-12

**Authors:** Jianhui Qiu, Ruiyi Deng, Zihou Zhao, Peidong Tian, Jingcheng Zhou

**Affiliations:** 1 Department of Urology, Peking University First Hospital, No. 8 Xishiku Street, Xicheng District, Beijing, 100034 People’s Republic of China; 2 Institute of Urology, Peking University, Beijing, China; 3 National Urological Cancer Center, Beijing, China

**Keywords:** Partial nephrectomy, Local tumor destruction, Non-clear cell renal cell carcinoma, Overall survival, Cancer-specific survival

## Abstract

**Purpose:**

There is a lack of authoritative opinions on local tumor destruction (LTD) for clinical T1a (cT1a) non-clear cell renal cell carcinoma (nccRCC). We aim to compare the outcomes of cT1a nccRCC after partial nephrectomy (PN) or LTD and explore prognostic factors.

**Methods:**

Patients diagnosed with cT1a nccRCC receiving LTD or PN between 2000 and 2020 were identified from the Surveillance, Epidemiology, and End Results (SEER) database. A 1:1 propensity score matching (PSM) was performed for patients receiving LTD and PN. Kaplan–Meier survival analysis, Cox regression analysis, competing risk regression models, and subgroup analysis were used to compare outcomes and identify prognostic factors. Prognostic nomograms were established and evaluated based on the multivariate models.

**Results:**

A total of 3664 cT1a nccRCC patients were included. The LTD group had poorer overall survival (OS) and similar cancer-specific survival (CSS) compared with the PN group before and after PSM (p < 0.05), while the other-cause mortality rate of the LTD group was higher than that of the PN group. Age, marital status, household income, prior tumor history, interval between diagnosis and treatment, treatments, and tumor size were identified as independent predictive factors for OS. Age, tumor size, prior tumor history, and histological type were identified as independent predictive factors for CSS. Then the nomograms predicting OS and CSS were constructed based on these prognostic factors, which showed excellent performance in risk stratification and accuracy.

**Conclusion:**

LTD could achieve comparable cancer-control effects as PN among cT1a nccRCC patients. The OS and CSS nomograms worked effectively for prognosis assessment.

**Supplementary Information:**

The online version contains supplementary material available at 10.1007/s00432-023-05571-8.

## Introduction

Renal cell carcinoma (RCC) is one of the most common malignancies worldwide with an incidence rate of 4.6/100000 in 2020, and the incidence rate has been escalating in recent decades (Miller et al. 2022; Bukavina et al. 2022). RCC comprises a heterogeneous group of malignancies that have characteristic histologic features with distinct genetic profiles and biological behaviors (Cancer Genome Atlas Research Network 2013; Davis et al. 2014; Cancer Genome Atlas Research Network et al. 2016). Clear cell RCC (ccRCC) accounts for 75–85% of all RCC, followed by papillary RCC (pRCC, 10–15%), chromophobe RCC (chRCC, 5–10%) and other rare tumors (Dudani et al. 2021). With the developments of imaging technology, RCC is increasingly being diagnosed early as low-stage incidental findings (Ljungberg et al. 2022). Most of the incidental renal masses were small renal masses (SRMs), of which diameters were less than 4 cm, making up 48–66% of all renal tumors (Sanchez et al. 2018). Partial nephrectomy (PN) could preserve more renal function without compromising oncological control than radical nephrectomy (RN) for clinical T1a (cT1a) RCC, thus it was recommended as the standard treatment for cT1a RCC by authoritative guidelines (Campbell et al. 2021). However, PN comes with a high surgical complication rate as high as 20%, such as bleeding and infection, which means it is not suitable for patients with advanced age and/or with severe comorbidities (Junker et al. 2022).

In recent years, local tumor destructions (LTDs), mainly representing cryoablation and thermal ablation, were widely adopted as alternative treatments in cT1a RCC patients unfit or unacceptable for PN (Ha et al. 2015; Shi et al. 2020), as LTD could offer a minimally invasive procedure with low risks of complications (Thompson et al. 2015; Lehrer et al. 2023). However, there are still discrepancies in the usage of LTD for cT1a RCC (Campbell et al. 2021; Ljungberg et al. 2022). Although some studies with small sample sizes reported that LTD was an effective and safe therapy for cT1a RCC, there is a lack of high-quality evidence to support LTD as a standard treatment (Shi et al. 2020). And most clinical trials and attempts about LTDs for SRMs were mainly based on ccRCC patients leading to the neglect of non-clear cell RCC (nccRCC). Limited by the low incidence, there was little progress in therapeutic options for nccRCC (Garje et al. 2021).

There exists some evidence that oncological outcomes for RCC differ based on histologic types which should be considered in the treatment decision-making process. A study including 229 patients with cT1a RCC (mean diameter 2.5 cm) treated by LTD, the disease-free survival at 80 months of pRCC was higher than that of ccRCC (100% vs. 87%, p = 0.04) (Lay et al. 2015). Another study comparing LTD and PN reported worse outcomes with LTD vs. PN in cT1b ccRCC. However, no significant difference in outcomes was discovered in nccRCC patients (Liu et al. 2017). Given the heterogeneity among RCCs and the lack of high-quality evidence, it’s necessary to investigate the effects of LTD for cT1a nccRCC patients.

The Surveillance, Epidemiology, and End Results (SEER) database summarizes cancer incidence information from 17 registries covering about half of the United States population (https://seer.cancer.gov/data/), based on which we conducted this population-based study to compare the prognosis of cT1a nccRCC patients after PN or LTD and explore prognostic factors. Nomograms have been widely used in oncology fields to estimate individualized risk when making treatment decisions (Balachandran et al. 2015; Wu et al. 2020). In this research, nomograms were subsequently developed to predict the prognosis of cT1a nccRCC patients receiving nephron-sparing treatments. This study could provide evidence for the optimization of the current treatment paradigm for cT1a nccRCC.

## Material and methods

### Study population

Patients diagnosed with renal tumors (Site record ICD-O-3 2023 Revision: Kidney Parenchyma) between January 1, 2000 and December 31, 2020 were identified. The inclusion criteria were as follows: (1) cT1a (≤ 4 cm) nccRCC without lymph node involvement and distant metastasis (cT1aN0M0); (2) receiving PNs or LTDs. LTDs mean cryosurgery, thermal ablation, electrocautery, laser ablation, etc. Patients with unavailable key demographic or tumor clinicopathological information were excluded. The eligible subjects were randomized 7:3 into the training cohort and the validation cohort. The training set was used to develop nomograms and the validation set was used for external validation.

### Clinical characteristics and outcome measurement

Demographic and oncological information was exported, such as patient ID, age at diagnosis, year of diagnosis, gender, race, marital status, median household income, rural/urban population density, tumor laterality, American Joint Committee on Cancer (AJCC) TNM stage, tumor size, histological types, histological grades, surgical treatments, survival status, survival time, cause of death, etc. Some variables were regrouped, including age at diagnosis (< 65, 65–85, > 85), race (white, black, others), marital status, median household income ($ 0–75,000, $ 75,000 +), prior tumor number (0, 1, ≥ 2), tumor size (< 3 cm, 3–4 cm), histological grades (G1&G2, G3&G4), and histologic types (pRCC, chRCC, cyst-associated RCC, others). All-cause mortality (ACM), cancer-specific mortality (CSM) and other cause mortality (OCM) according to the SEER registry were defined. The follow-up period was defined as the time from diagnosis to death, loss to follow-up, or end of study. The primary endpoint and the secondary endpoint were overall survival (OS) and cancer-specific survival (CSS). Censoring occurred at the end of the available follow-up unless the occurrence of CSM or OCM.

### Statistical analysis

Eligible subjects were divided into the PN group and the LTD group according to the treatment status. 1:1 propensity score matching (PSM) according to the nearest neighbor was adopted to optimize the comparison between groups and reduce the influence of potential confounding factors (Austin 2011). Age, gender, race, marital status, median household income, rural/urban population density, laterality, prior tumor history, the time interval between diagnosis and treatments, tumor size, grades, and histologic types were used for calculating propensity score (PS) in a multivariate logistic regression manner for each patient. The process was conducted using the “MatchIt” package in R software with a caliper width of 0.002.

All demographic and clinicopathologic characteristics were reported using descriptive statistics. Frequencies and proportions were reported for categorical variables. Mean and standard deviation (SD) were reported for continuous variables. The intergroup difference of baseline characteristics was compared with the student’s test, the Chi-square, or Mann–Whitney *U* test as appropriate. Differences in OS and CSS between groups were compared via Kaplan–Meier (KM) analysis and stratified log-rank tests. Univariate and multivariate Cox regression analysis models were employed to identify the variables that significantly impact OS. Considering that OCM could be competing events to CSM, KM or Cox analysis may not be the best choices because these two methods regard competing events as independent censored events and overrate the incidence of target events. Instead, competing risk regression models (CRR) based on the Fine-Gray regression could discriminate the effects of clinical factors on special events (Zhang 2017). Thus, CRR was used to assess the predictive factors of CSM. Cumulative incidence plots were used to illustrate CSM and OCM. Variables with p-value < 0.1 in univariate models were included in further multivariate models when conducting both Cox regression analysis and CRR analysis. Moreover, subgroup analysis was performed to probe the survival impacts of different treatments in each subgroup. The forest maps of OS and CSS for visualization were conducted by the “forestplot” package.

The factors potentially influencing the prognosis of cT1a nccRCC patients receiving nephron-sparing treatments were incorporated in the least absolute shrinkage and selection operator (LASSO) regression analysis to identify useful predictive factors, which could avoid overfitting to some extent and select the best weighting coefficient of clinical characteristics (Tibshirani 1997; Tang et al. 2021). The nomograms predicting OS and CSS were developed using the “rms” package in R. The inclusion of covariates in the nomograms followed Harrell’s guideline (Balachandran et al. 2015). KM analysis and CRR were employed to evaluate the ability of risk stratification. The Harrell’s concordance index (C-index) was calculated to evaluate discriminative ability. Calibration curves were applied to verify the consistency between the predicted values and the actual results. Moreover, decision curve analyses (DCAs) were to depict the clinical net benefit and utility of the nomograms at different risk threshold probabilities (Van Calster et al. 2018).

All statistical tests were performed using the R software (version 4.3.1). A two-sided with P < 0.05 was considered to be indicative of statistical significance. All analyses were performed according to the STROBE statement (von Elm et al. 2007).

## Results

### Descriptive characteristics of the population

**Fig. 1:**
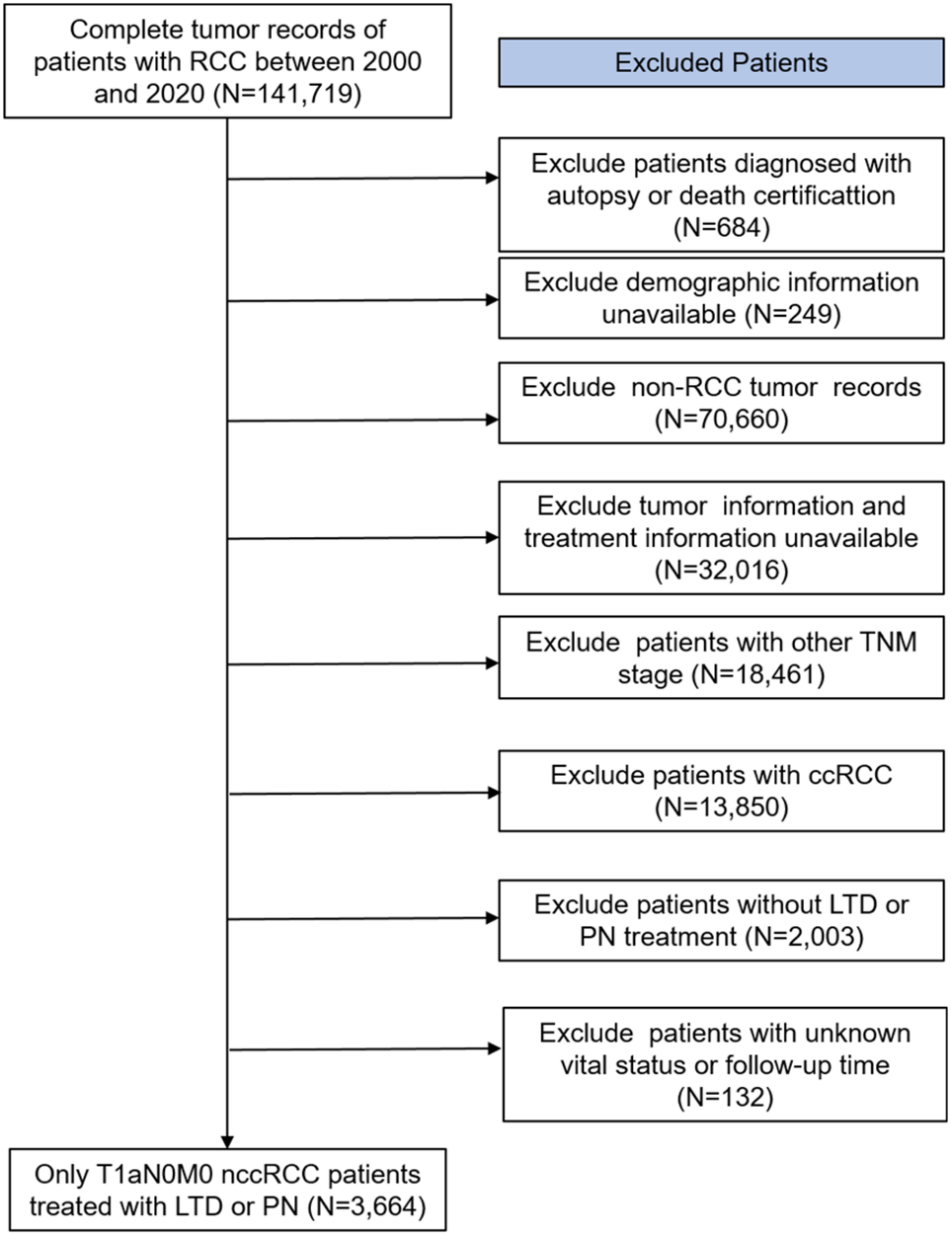
The flow chart of study participant selection

As shown in Fig. 1, 3664 patients with nccRCC accepting LTD (n = 690) or PN (n = 2974) were included. Baseline characteristics of patients in the LTD and the PN groups are shown in Table 1. The median [interquartile range/IQR] age at diagnosis for the overall cohort was 66 [59, 72] years. The median follow-up period for the overall cohort, the LTD group, and the PN group was 89, 66, and 92 months, respectively. In LTD group, 454 patients accepted cryosurgery, 176 patients accepted thermal ablation, and 60 patients accepted other LTD treatments. Compared with the PN group, patients in the LTD group were older (> 65 years: 72.7% vs 51.1%, p < 0.001), had less proportion of 3–4 cm tumor (3–4 cm, 21.2% vs 26.8%, p = 0.005), longer time interval between diagnosis and treatment [mean ± SD] (1.72 ± 2.52 months vs 1.15 ± 2.13 months, p < 0.001), and a larger proportion of the prior tumor history (70.1% vs 59.7%, p < 0.001). Most of the histologic grades (56.1%) in PN groups were I&II, whereas 61.2% of the grade in LTD groups were unknown/inapplicable. The most common histologic type was pRCC (76.0% in the overall cohort, 81.9% in LTD group, and 74.7% in PN group), followed by chRCC (20.6% in the overall cohort, 16.4% in LTD group, and 21.6% in PN group). No statistically significant differences were discovered for gender, race, and marital status (p > 0.05). After PSM, 435 patients in each group were identified, and all clinical characteristics between the two groups were well balanced (p > 0.05). The distribution of PS and the histogram of PS indicated that the equilibrium of baseline characteristics had been reached between the LTD and the PN groups (Fig S1).

**Table 1:** Baseline characteristics of T1a non-ccRCC patients between LTD and PN groups before and after PSM

Characteristics	Before PSM				After PSM			
	Overall (n = 3664)	LTD (n = 690)	PN (n = 2974)	p-value^a^	Overall (n = 870)	LTD (n = 435)	PN (n = 435)	p-value
Age, n (%)								
< 65	1640 (44.8)	187 (27.1)	1453 (48.9)	** < 0.001**	318 (36.6)	155 (35.6)	163 (37.5)	0.13
65–85	1973 (53.8)	464 (67.2)	1509 (50.7)		545 (62.6)	279 (64.1)	266 (61.1)	
> 85	51 (1.4)	39 (5.7)	12 (0.4)		7 (0.8)	1 (0.2)	6 (1.4)	
Gender, n (%)								
Female	895 (24.4)	160 (23.2)	735 (24.7)	0.429	209 (24.0)	97 (22.3)	112 (25.7)	0.267
Male	2769 (75.6)	530 (76.8)	2239 (75.3)		661 (76.0)	338 (77.7)	323 (74.3)	
Race, n (%)								
White	2793 (76.2)	540 (78.3)	2253 (75.8)	0.371	653 (75.1)	333 (76.6)	320 (73.6)	0.572
Black	754 (20.6)	129 (18.7)	625 (21.0)		190 (21.8)	90 (20.7)	100 (23.0)	
Others	117 (3.2)	21 (3.0)	96 (3.2)		27 (3.1)	12 (2.8)	15 (3.4)	
Marital status, n (%)								
None/unknown	1254 (34.2)	236 (34.2)	1018 (34.2)	1	290 (33.3)	145 (33.3)	145 (33.3)	1
Yes	2410 (65.8)	454 (65.8)	1956 (65.8)		580 (66.7)	290 (66.7)	290 (66.7)	
Median household income, n (%)								
$ (0–75,000)	2036 (55.6)	372 (53.9)	1664 (56.0)	0.353	466 (53.6)	235 (54.0)	231 (53.1)	0.838
$ (75,000 +)	1628 (44.4)	318 (46.1)	1310 (44.0)		404 (46.4)	200 (46.0)	204 (46.9)	
Rural/urban population density, n (%)								
Counties in metropolitan areas ge 1 million pop	2360 (64.4)	434 (62.9)	1926 (64.8)	0.399	558 (64.1)	279 (64.1)	279 (64.1)	0.8
Counties in metropolitan areas of 0 to 1 million pop	973 (26.6)	198 (28.7)	775 (26.1)		239 (27.5)	117 (26.9)	122 (28.0)	
Nonmetropolitan counties	327 (8.9)	58 (8.4)	269 (9.0)		73 (8.4)	39 (9.0)	34 (7.8)	
Unknown	4 (0.1)	0 (0.0)	4 (0.1)		0 (0.0)	0 (0.0)	0 (0.0)	
Laterality, n (%)								
Left	1818 (49.6)	329 (47.7)	1489 (50.1)	0.277	414 (47.6)	207 (47.6)	207 (47.6)	1
Right	1846 (50.4)	361 (52.3)	1485 (49.9)		456 (52.4)	228 (52.4)	228 (52.4)	
Prior tumor number, n (%)								
0	1405 (38.3)	206 (29.9)	1199 (40.3)	** < 0.001**	299 (34.4)	152 (34.9)	147 (33.8)	0.733
1	1838 (50.2)	366 (53.0)	1472 (49.5)		447 (51.4)	225 (51.7)	222 (51.0)	
> 2	421 (11.5)	118 (17.1)	303 (10.2)		124 (14.3)	58 (13.3)	66 (15.2)	
Interval between diagnosis and treatment (month, mean [SD])	1.26 [2.22]	1.72 [2.52]	1.15 [2.13]	** < 0.001**	1.33 [2.21]	1.33 [1.99]	1.33 [2.41]	0.174
Tumor size, n (%)								
< 1 cm	131 (3.6)	16 (2.3)	115 (3.9)	**0.005**	25 (2.9)	14 (3.2)	11 (2.5)	0.743
1–2 cm	1160 (31.7)	234 (33.9)	926 (31.1)		291 (33.4)	139 (32.0)	152 (34.9)	
2–3 cm	1430 (39.0)	294 (42.6)	1136 (38.2)		377 (43.3)	190 (43.7)	187 (43.0)	
3–4 cm	943 (25.7)	146 (21.2)	796 (26.8)		177 (20.3)	92 (21.1)	85 (19.5)	
Histologic grade, n (%)								
Grade I	425 (11.6)	86 (12.5)	339 (11.4)	** < 0.001**	133 (15.3)	65 (14.9)	68 (15.6)	0.841
Grade II	1491 (40.7)	161 (23.3)	1330 (44.7)		276 (31.7)	145 (33.3)	131 (30.1)	
Grade III	610 (16.6)	20 (2.9)	590 (19.8)		45 (5.2)	20 (4.6)	25 (5.7)	
Grade IV	35 (1.0)	1 (0.1)	34 (1.1)		2 (0.2)	1 (0.2)	1 (0.2)	
Unknown/unapplicable	1103 (30.1)	422 (61.2)	681 (22.9)		414 (47.6)	204 (46.9)	210 (48.3)	
Histologic type, n (%)								
pRCC	2786 (76.0)	565 (81.9)	2221 (74.7)	**0.001**	666 (76.6)	343 (78.9)	323 (74.3)	0.438
chRCC	756 (20.6)	113 (16.4)	643 (21.6)		188 (21.6)	85 (19.5)	103 (23.7)	
Cyst-associated RCC	95 (2.6)	8 (1.2)	87 (2.9)		9 (1.0)	4 (0.9)	5 (1.1)	
Sarcomatoid RCC	19 (0.5)	2 (0.3)	17 (0.6)		4 (0.5)	1 (0.2)	3 (0.7)	
Collecting duct RCC	8 (0.2)	2 (0.3)	6 (0.2)		3 (0.3)	2 (0.5)	1 (0.2)	

^a^p-value < 0.05 will be bold

### Survival analysis of the treatment groups

**Fig. 2:**
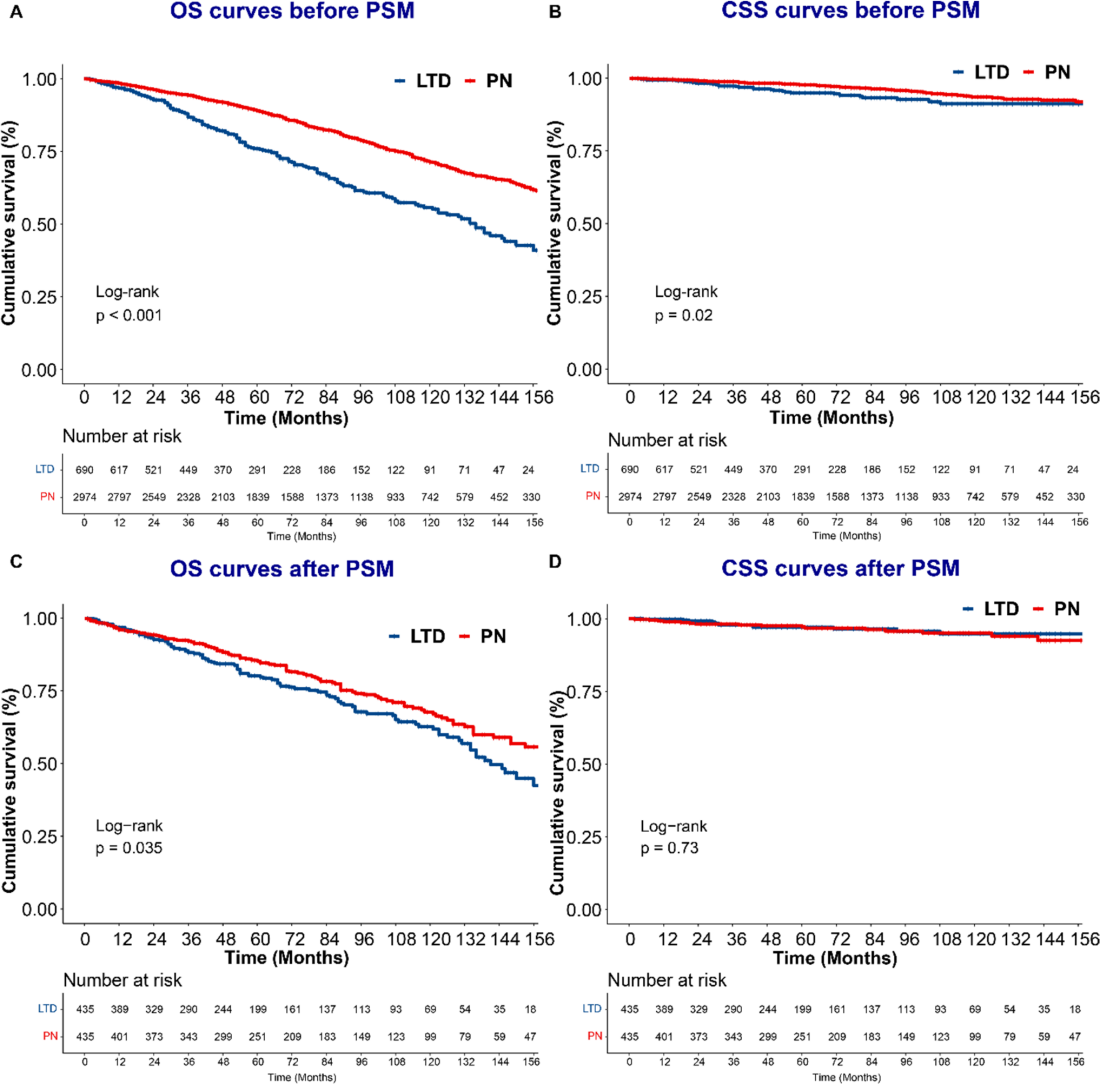
The Kaplan‒Meier curves of OS (**A** and **C**) and CSS (**B** and **D**) for T1a non-ccRCC patients stratified by treatments before or after PSM

**Fig. 3:**
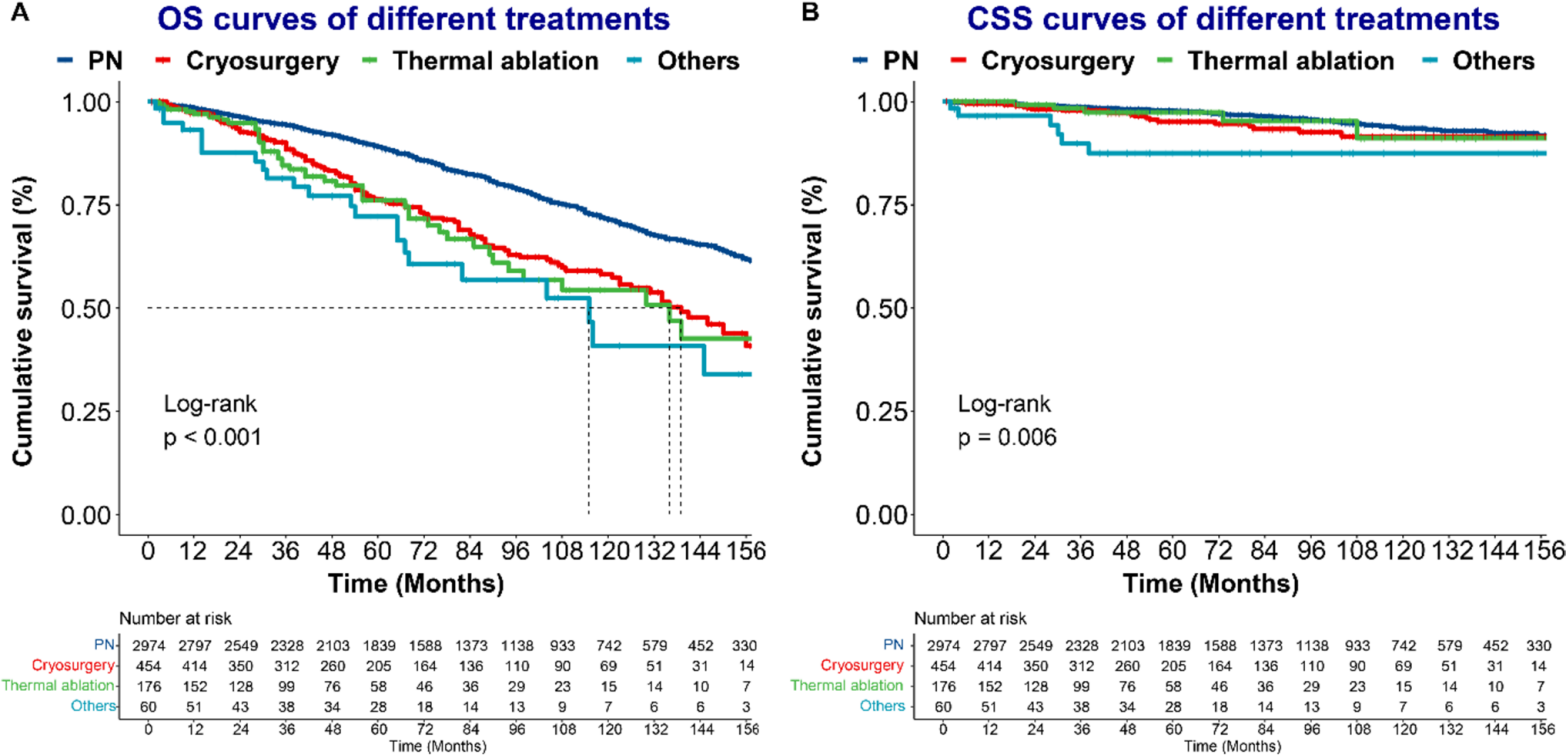
The Kaplan‒Meier curves of OS (**A**) and CSS (**B**) for T1a non-ccRCC patients stratified by specific treatments

KM analysis was performed among nccRCC patients before and after PSM. The 3-, 5-, and 10- OS rates for all patients were 93.0%, 86.8%, 68.8%. The 3-, 5-, and 10- CSS rates for all patients were 98.5%, 97.3%, 93.2%. Compared with PN, LTD correlated with worse OS (3-year OS rate: 86.9% vs. 94.3%, 5-year OS rate: 76.0% vs. 89.0%, 10-year OS rate: 55.8% vs. 71.4%, p < 0.001; Fig. 2A) and worse CSS (3-year CSS rate: 97.3% vs. 98.8%, 5-year CSS rate: 95.0% vs. 97.8%, 10-year CSS rate: 91.2% vs. 93.6%, p = 0.02; Fig. 2B) in all populations before PSM. After PSM, patients in the LTD group still had worse OS than those in the PN group (3-year OS rate: 88.2% vs. 92.2%, 5-year OS rate: 80.2% vs. 85.3%, 10-year OS rate: 62.8% vs. 67.8%, p = 0.035) (Fig. 2C, Table S1). However, there was no significant difference in CSS between LTD and PN groups after PSM (3-year CSS rate: 97.9% vs. 98.3%, 5-year CSS rate: 97.2% vs. 97.3%, 10-year CSS rate: 94.7% vs. 95.1%, p = 0.73) (Fig. 2D). Regarding specific treatments, PN correlated with the best OS among all patients (Fig. 3A). Patients receiving cryosurgery and thermal ablation had similar OS (Table S2). Similar long-term CSS was observed in patients receiving PN, cryosurgery, and thermal ablation (Fig. 3B, Table S2).

### Evaluation of prognostic factors for OS and CSS

Potential prognostic factors associated with OS were included in univariate and multivariate Cox analysis before PSM. Age at diagnosis, marital status, household income, the history of prior tumors, the time interval between diagnosis and treatment, treatments for nccRCC, tumor size, and histologic grade were identified as potential independent predictors for OS in the univariate Cox analysis (p < 0.05) (Table 2). In the multivariate Cox analysis, older age (65–85 years, hazard ratio/HR = 2.16, 95% confidence interval/CI, 1.86–2.52, p < 0.001; > 85 years, HR = 5.99, 95% CI, 3.99–9.00, p < 0.001), unmarried status (married status, HR = 0.80, 95%CI 0.70–0.92, p = 0.002), lower household income (> $ 75,000, HR = 0.85, 95%CI, 0.74–0.98, p = 0.025), ≥ 2 prior tumors (HR = 1.80, 95%CI, 1.47–2.22, p < 0.001), longer time interval between diagnosis and treatment (per month, HR = 1.04, 95% CI, 1.01–1.07, p = 0.015), LTD treatment (HR = 1.52, 95% CI, 1.27–1.82, p < 0.001), and larger tumor size (3-4 cm, HR = 1.24, 95% CI, 1.07–1.44, p = 0.005) were identified as independent risk factors for OS (Table 2).

**Table 2:** Univariate and multivariate Cox analysis for OS

Characteristics	Univariate analysis			Multivariate analysis		
	HR	95%CI	P-value^a^	HR	95%CI	P-value
Age groups (y)						
< 65	Reference			Reference		
65–85	2.32	2.00–2.69	** < 0.001**	2.16	1.86–2.52	** < 0.001**
> 85	8.66	5.88–12.8	** < 0.001**	5.99	3.99–9.00	** < 0.001**
Gender						
Female	Reference					
Male	0.96	0.82–1.12	0.587			
Race						
White	Reference					
Black	1.02	0.86–1.20	0.850			
Others	0.71	0.44–1.16	0.171			
Marital status						
None/unknown	Reference			Reference		
Married	0.82	0.71–0.94	**0.005**	0.80	0.70–0.92	**0.002**
Median household income						
$(0–75,000)	Reference			Reference		
$(75,000 +)	0.86	0.75–0.99	**0.032**	0.85	0.74–0.98	**0.025**
Rural/urban population density						
Counties in metropolitan areas ge 1 million pop	Reference					
Counties in metropolitan areas of 0 to 1 million pop	1.03	0.88–1.20	0.724			
Nonmetropolitan counties	1.10	0.87–1.39	0.869			
Laterality						
Left	Reference					
Right	0.92	0.81–1.06	0.242			
Prior tumor number						
0	Reference			Reference		
1	1.09	0.94–1.26	0.075	1.00	0.86–1.16	0.972
≥ 2	2.28	1.86–2.79	** < 0.001**	1.80	1.47–2.22	** < 0.001**
Interval between diagnosis and treatment (per month)	1.05	1.02–1.08	**0.001**	1.04	1.01–1.07	**0.015**
Treatment						
PN	Reference			Reference		
LTD^b^	1.97	1.68–2.31	< **0.001**	1.52	1.27–1.82	** < 0.001**
LTD^c^	1.33	1.02–2.72	**0.036**			
Tumor size						
< 3 cm	Reference			Reference		
3–4 cm	1.21	1.04–1.40	**0.014**	1.24	1.07–1.44	**0.005**
Histologic grade						
G1 & G2	Reference					
G3 & G4	1.03	0.85–1.25	0.745			
Unknown/unapplicable	1.26	1.08–1.47	**0.003**			
Histologic type						
pRCC	Reference					
chRCC	0.95	0.81–1.13	0.582			
Cyst-associated RCC	0.82	0.55–1.22	0.333			
Others	1.07	0.56–2.07	0.834			

^a^p-value < 0.05 will be bold

^b^Univariate analysis before PSM

^c^Univariate analysis after PSM

**Fig. 4:**
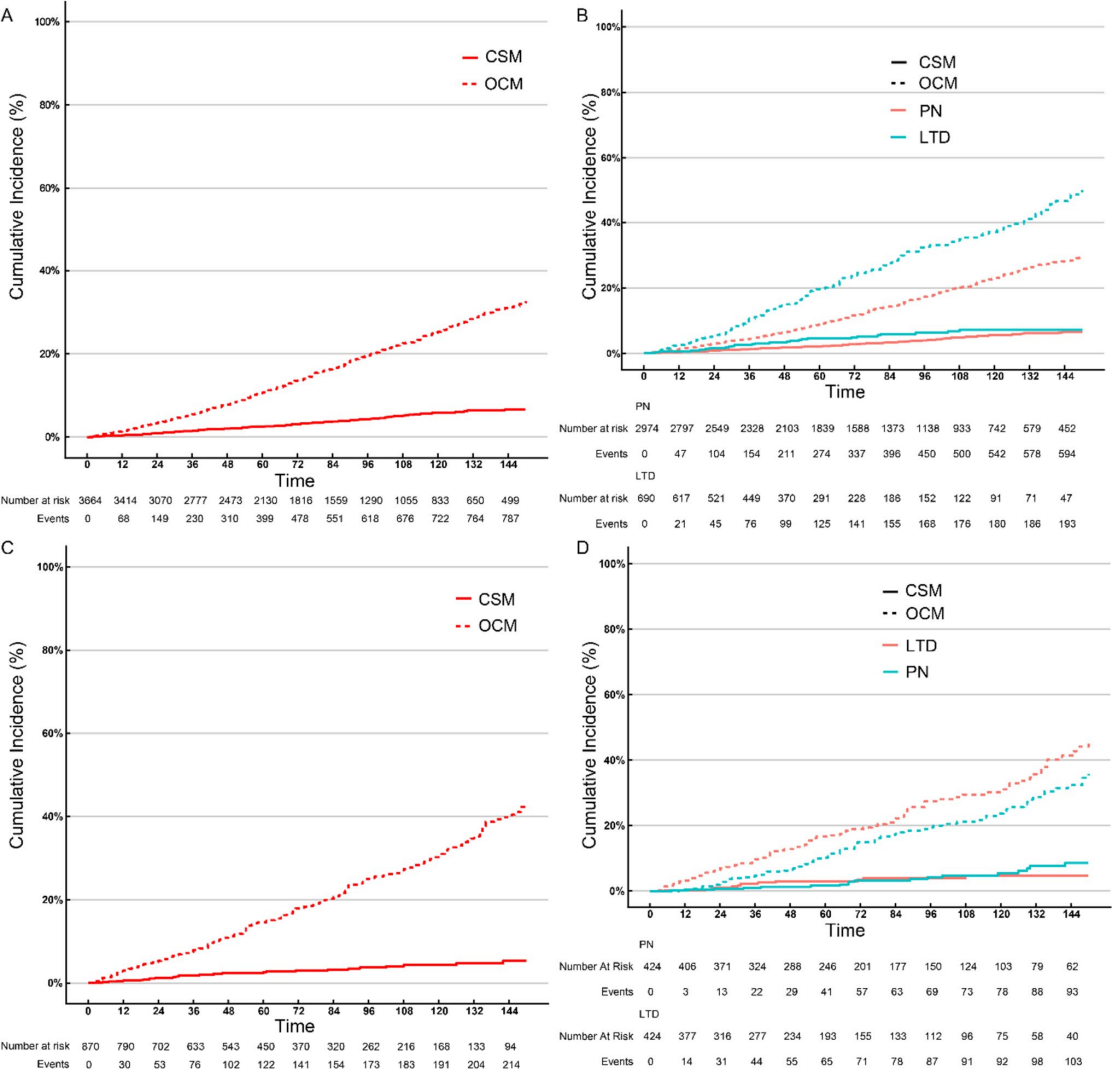
Cumulative incidence plots of CRR models in overall cohort before (**A**) or after (**C**) PSM, and stratified by treatments before (**B**) or after (**D**) PSM

The 3-, 5-, and 10-year CSM rates in the overall cohort were 1.45%, 2.55%, and 5.90%, and the 3-, 5-, and 10-year OCM rates in the overall cohort were 5.53%, 10.7%, and 25.3%, respectively (Fig. 4A). The cumulative incidence plots showed that the 3-, 5-, and 10-year OCM rates of the LTD group (10.6%, 19.5%, 37.1%) were higher than that of the PN group (4.45%, 8.86%, 23.0%). However, the 3-, 5-, and 10-year CSM rates didn’t show significant difference between the LTD group (2.55%, 4.53%, 7.18%) and the PN group (1.21%, 2.13%, 5.62%, Fig. 4B, p = 0.120). LTD correlated with similar CSS with PN even after PSM (Fig. 4C-D, p = 0.727).

The results of univariable CRR demonstrated that age, prior tumor number, grade, and histologic type were potential independent predictors for CSS. Compared with PN, receiving LTD didn’t lead to significant difference in CSM before (subdistribution hazard ratio/SHR = 1.37, 95% CI, 0.92–2.03, p = 0.120) and after PSM (SHR = 0.88, 95% CI, 0.42–1.84, p = 0.727). Further multivariable CRR showed that older age (65–85 years, SHR = 1.43, 95% CI, 0.98–2.01, p = 0.044; > 85 years, SHR = 3.33, 95% CI, 1.29–8.57, p = 0.013), a history of prior tumor (≥ 2, SHR = 2.41, 95% CI, 1.51–3.87, p < 0.001), larger tumor size (3-4 cm, SHR = 1.51, 95% CI, 1.05–2.16, p = 0.026), and other nccRCC histological type (SHR = 2.91, 95% CI, 1.11–7.64, p = 0.031) were independent risk factors for CSM (Table 3).

**Table 3:** Univariate and multivariate competing-risk analysis for CSS

	Univariate analysis			Multivariate analysis		
	HR	95%CI	P-value^a^	HR	95%CI	P-value
Age groups (y)						
< 65	Reference			Reference		
65–85	1.58	1.13–2.21	**0.007**	1.43	1.01–2.02	**0.044**
> 85	3.63	1.43–9.19	**0.008**	3.33	1.29–8.57	**0.013**
Gender						
Female	Reference					
Male	1.24	0.83–1.84	0.300			
Race						
White	Reference					
Black	1.08	0.73–1.60	0.700			
Others	0.23	0.03–1.67	0.150			
Marital status						
None/unknown	Reference					
Married	1.21	0.85–1.72	0.290			
Median household income						
$(0–75,000)	Reference			Reference		
$(75,000 +)	0.75	0.54–1.05	0.092	0.76	0.54–1.07	0.120
Rural/urban population density						
Counties in metropolitan areas ge 1 million pop	Reference					
Counties in metropolitan areas of 0 to 1 million pop	0.74	0.50–1.11	0.140			
Nonmetropolitan counties	1.21	0.73–2.02	0.460			
Laterality						
Left	Reference			Reference		
Right	0.72	0.52–1.02	0.055	0.74	0.53–1.03	0.070
Prior tumor number						
0	Reference			Reference		
1	1.43	1.00–2.03	**0.048**	1.39	0.97–2.00	0.073
≥ 2	2.66	1.69–4.18	** < 0.001**	2.41	1.51–3.87	** < 0.001**
Interval between diagnosis and treatment (per month)	0.96	0.88–1.05	0.360			
Treatment						
PN	Reference					
LTD^b^	1.37	0.92–2.03	0.120			
LTD^c^	0.88	0.42–1.84	0.727			
Tumor size						
< 3 cm	Reference			Reference		
3–4 cm	1.37	0.97–1.92	0.074	1.51	1.05–2.16	**0.026**
Histologic grade						
G1 & G2	Reference					
G3 & G4	1.16	0.74–1.81	0.530			
Unknown/unapplicable	1.45	1.02–2.07	**0.040**			
Histologic type						
pRCC	Reference			Reference		
chRCC	0.77	0.50–1.18	0.230	0.75	0.49–1.15	0.180
Cyst-associated RCC	0.58	0.18–1.83	0.350	0.63	0.20–1.96	0.420
Others	2.90	1.09–7.67	**0.032**	2.91	1.11–7.64	**0.031**

^a^p-value < 0.05 will be bold

^b^Univariate analysis before PSM

^c^Univariate analysis after PSM

**Fig. 5:**
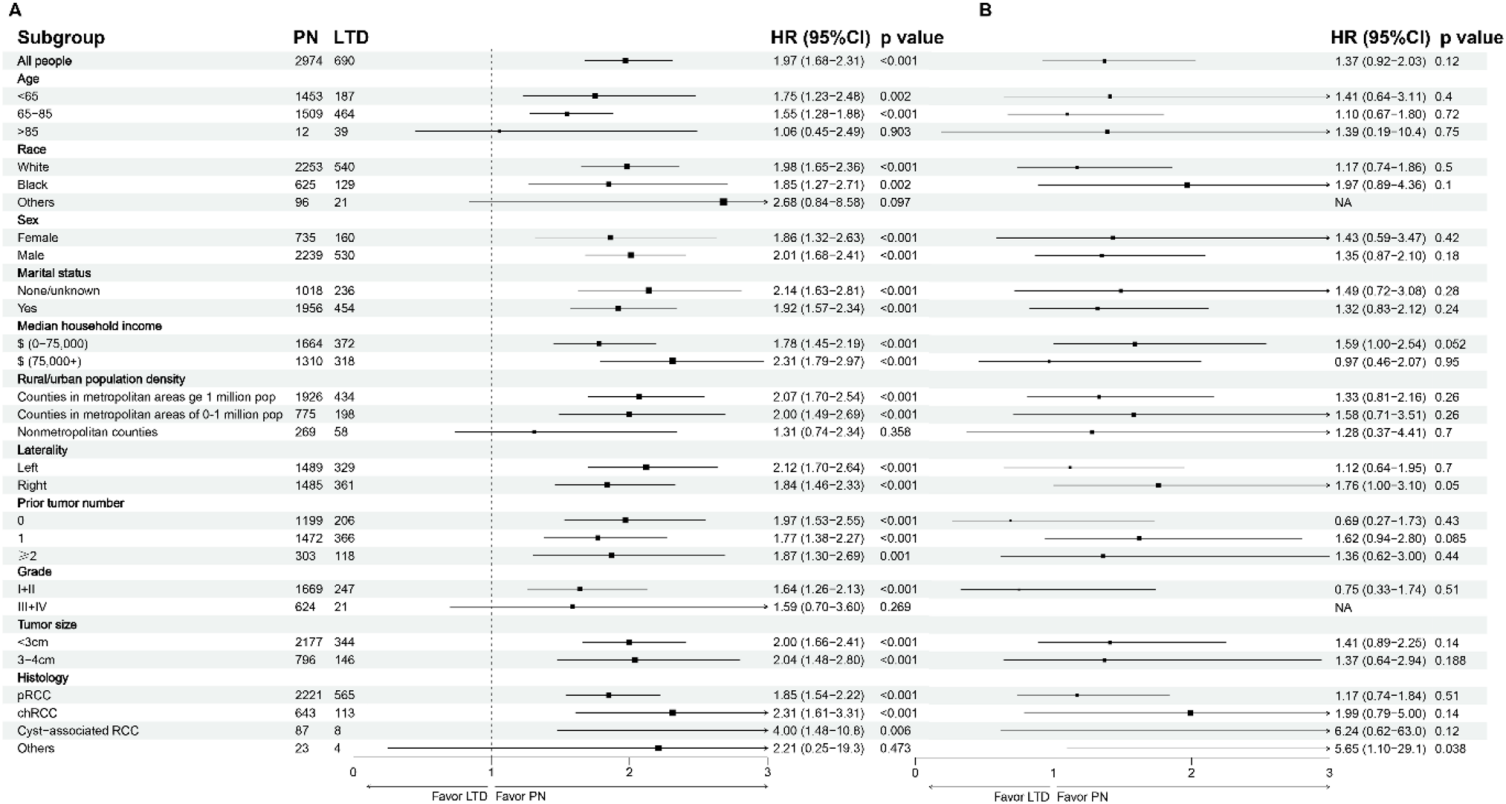
Forest plot of subgroup analysis for OS (A) and CSS (B)

To probe the impacts of treatments on the survival of nccRCC patients and reduce the influence of potential confounding factors, subgroup analyses were conducted among patients in the overall cohort (Fig. 5). LTD was associated with worse OS in most of the subgroups (HR > 1, p < 0.05). There was no significant difference between LTD and PN in subgroups of patients older than 85 (HR = 1.06, 95% CI, 0.45–2.49, p = 0.903). Patients with cT1a nccRCC accepting LTD or PN had similar CSS in most subgroups, consistent with the result in the overall cohort. Limited by the low rate of CSM, some subgroup analyses for CSS were inapplicable.

### Development and validation of nomograms

**Fig. 6:**
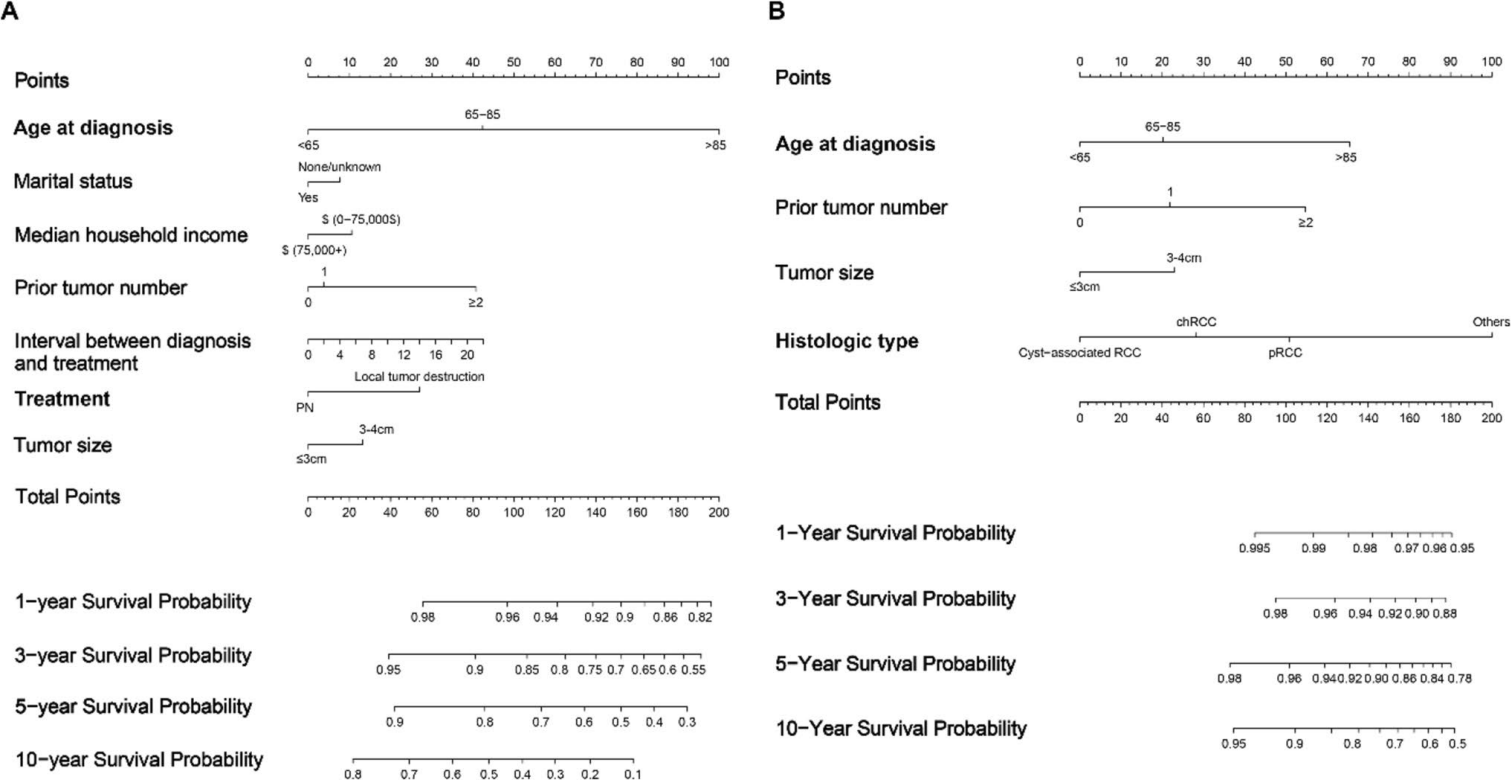
Nomograms for predicting the 1-, 3-, 5-, and 10-year OS (A) and CSS (B) of cT1a nccRCC patients receiving nephron-sparing treatments

The cT1a nccRCC patients receiving nephron-sparing treatments were randomly divided into the training cohort (n = 2564) and the validation cohort (n = 1100). The baseline demographic and clinicopathological characteristics of patients in the training cohort and the validation cohort were well-balanced (Table S3). To further investigate the potential prognostic factors identified by multivariable Cox analysis and multivariable CRR models, LASSO regression analysis was employed. Age at diagnosis, marital status, household income, prior tumor number, interval between diagnosis and treatments, treatments and tumor size were included in the LASSO regression analysis to identify risk factors of OS (Fig S2A, B). The optimal λ value of 0.0015 was obtained through cross-validation. According to the LASSO regression analysis, the seven variables mentioned above were finally incorporated into the OS nomogram (Fig. 6A). Age at diagnosis, prior tumor number, tumor size, and histologic typewere included in the LASSO regression analysis. Histologic grade was not included because only the p-value of unknown/inapplicable type was below 0.1. Eventually, the optimal λ value was 0.0033 (Fig S2C, D). The nomogram for CSS was developed based on the CRR model and the LASSO regression (Fig. 6B).

The median [IQR] risk score of OS was 66 [30–89], based on which patients were divided into low- and high-risk subgroups. Significant difference in OS was observed between the two subgroups in the training cohort (Fig S3A) and the validation cohort (Fig S3B). The C-index was 0.759 [95%CI: 0.734–0.784] in the training cohort and 0.774 [95%CI: 0.739–0.809] in the validation cohort, indicating favorable discrimination by the OS nomogram. Then calibration plots were generated for 1-, 3-, 5-, and 10-year OS in the training cohort and the validation cohort, which showed high consistencies between the predicted probabilities and the actual survival outcomes (Fig S4). Furthermore, DCA curves showed that the nomogram could well predict the probability of OS, as it added more net benefits compared with both the treat-all-patients scheme and the treat-none scheme. The OS nomogram resulted in greater net benefit across threshold probabilities between 10 and 70% for 5-year OS, and threshold probabilities between 15 and 95% for 10-year OS (Fig S6A–D). Compared with previous model for T1a RCC patients (Tang et al. 2023), the threshold probabilities of this predictive model displayed remarkable net benefits and better performance in predicting OS of patients with cT1a nccRCC.

As for CSS, the median [IQR] risk score was 107 [84–136]. There was significant difference between the two groups for CSM in the training cohort (**Fig S3C**, p < 0.001) and the validation cohort (**Fig S3D**, p = 0.047). The C-index was 0.769 [95%CI: 0.716–0.818] in the training cohort and 0.801 [95%CI: 0.731–0.871] in the validation cohort, indicating favourable discrimination by the CSS nomogram. The calibration plots for 1-, 3-, 5-, and 10-year CSS revealed a good consistency between the predicted and observed survival probability in the training cohort and validation cohort (**Fig S5**). The DCA curves showed that the CSS nomogram resulted in greater net benefit across threshold probabilities between 20 and 50% for 5-year CSS, and threshold probabilities between 60 and 90% for 10-year CSS (**Fig S6E-H**). The DCA curves based on our CSS predictive model exhibited relatively better performance in predicting long-term CSS of patients with cT1a nccRCC than the previous model (Sorce et al. 2023a).

## Discussion

Though PN has been deemed as the gold standard treatment for cT1a RCC, LTD was regarded as an alternative therapy to PN, especially for those with advanced age, multiple comorbidities, morbid obesity, or operation history of kidney (Motzer et al. 2022; Bertolotti et al. 2023). However, most published studies focused on ccRCC, not enough to support LTD on nccRCC (Talenfeld et al. 2018; Chan et al. 2022; Sorce et al. 2023a). nccRCC contains a heterogeneous group of malignancies, of which distinct biological behaviors could influence the therapeutic response and lead to different prognoses (Tang et al. 2023). Only a few studies focus on employing LTD for the treatment of nccRCC but were limited by the sample size and low incidence (Steffens et al. 2014; Nguyen et al. 2016; Shi et al. 2020). To clarify the gaps in this field, and help clinicians optimize the individual treatment strategies, we conducted this population-based study.

In this research, 3664 patients with cT1a nccRCC were included. Compared to patients in the PN group, those in the LTD group tended to be older and harbored smaller RCC. Advanced age is usually associated with more comorbidities, and a relatively frail state, predisposing to a higher probability of OCM, which goes some ways to explaining worse OS of LTD group (Sorce et al. 2023a; Pedraza-Sánchez et al. 2023).

For providing unbiased CSM risk estimates, CRR models were adopted relying on multivariable adjustments and adjusting for OCM. Correspondingly, no significant difference in CSS between the LTD group and the PN group was observed. Patients receiving PN or LTD had similar long-term CSS. These results indicated that LTD could achieve a comparable oncologic control effect as PN among cT1a nccRCC patients. Multivariable CRR showed that age at diagnosis, tumor size, prior tumor number, and histological types were independent predictive factors for CSM. The OCM rates in the overall cohort were higher than the corresponding CSM rates. A large attrition effect from high OCM rates may reduce the sample size of patients at risk of CSM, and shine a favorable light on CSM outcomes (Sorce et al. 2023b).

Previous studies demonstrated that LTD was associated with lower complication rates and shorter hospital stays than PN. LTD could avoid the clamping of the renal artery and surgical trauma, thus better preserving the kidney’s function more safely (Krokidis et al. 2018; Pedraza-Sánchez et al. 2023). In addition, the minimally invasive process of LTD improved the physical tolerability of elderly patients (Chan et al. 2022). Moreover, a shorter hospital stay is in line with health economics considerations (Garcia et al. 2021; Lehrer et al. 2023). Thus, LTD is a feasible alternative for elderly patients with/without comorbidities (Yan et al. 2019).

A higher proportion of patients older than 85 years in the LTD group may induce bias when evaluating OS. Further subgroup analysis stratified according to age showed that there was no significant difference in OS between LTD and PN among patients older than 85 years, whereas LTD was associated with significantly poor OS in most other subgroups. However, patients accepting LTD or PN shared similar CSS in most subgroups, even after a 10-year follow-up period. We supposed that the worse OS in the LTD group was due to the older age, higher Eastern Cooperative Oncology Group (ECOG) performance status scale, and more comorbidities of the LTD group compared to the PN group (Chan et al. 2022). This study didn’t contain these characteristics because of incomplete records in SEER registries.

Tumor size is an important parameter for a solid tumor and determining the treatment strategy, which has been identified as a risk factor for OS and CSS in this study (Palumbo et al. 2019; Shi et al. 2020). Johnson, B.A et al. demonstrated that RCCs bigger than 3 cm have been linked to a higher frequency of recurrence within the cT1a classification, with a disease-free survival rate of 68% compared to 97% of tumors < 3 cm (Johnson et al. 2019). Current authoritative guidelines advise that cT1a renal mass (< 3 cm) should be evaluated for renal thermal ablative therapies (Campbell et al. 2021; Bertolotti et al. 2023). A guideline published in 2022 supplemented that larger tumors of > 3–4 cm and those located at the hilum or near the proximal ureter should not be treated with ablative therapies (Ljungberg et al. 2022). However, the recommended tumor size cutoff values for LTD applications in nccRCC have not been specified in these guidelines (Motzer et al. 2022; Ljungberg et al. 2022; Bukavina et al. 2022). In subgroup analysis, the poor OS of patients treated with LTD was observed both in the ≤ 3 cm group and 3–4 cm group, while CSS after LTD or PN was similar. According to the results, we concluded that LTD achieved comparable oncological control effects for ≤ 3 cm and 3–4 cm nccRCC. Though the Fuhrman nuclear grade was an important prognostic factor in most studies, it was only available for ccRCC and pRCC (Paner et al. 2010). Therefore, the histologic grade had a restricted use for nccRCC and was not included in the final models in this study.

The OS and CSS for all patients, PN group, or LTD group in this study were higher than in previous studies (Andrews et al. 2019; Abdelsalam et al. 2023). It may be attributed to this study only incorporating nccRCC patients (76% pRCC, 20.6% chRCC), different from other studies including mainly ccRCC patients. It has been reported that the overall prognosis of nccRCC (mainly chRCC and pRCC) was better than ccRCC (Steffens et al. 2014; Kuthi et al. 2017). Some studies indicated that the prognosis of ccRCC after local ablation was worse than that of nccRCC (Lay et al. 2015; Liu et al. 2017). The mechanism of thermal ablation could partly explain these results. The generated heat energy tends to decrease near structures with continuous liquid flow, which has been known as the heat sink effect (Lay et al. 2015). High levels of vascularity in tumors would negatively affect thermal ablation effectiveness as blood flow could rapidly dissipate the heat energy (Liu et al. 2017). It has been widely known that ccRCC is a highly vascular tumor subtype, more so than pRCC and chRCC (Onishi et al. 2002). As such, there are biological and technical reasons to suppose that thermal ablation technologies may be more efficacious for nccRCC (Lay et al. 2015).

In recent years, the nomogram has been employed widely as a promising tool for prognosis prediction. Based on the potential prognostic factors identified by the multivariable Cox analysis and the multivariable CRR model, we conducted LASSO analysis and developed predictive nomograms for cT1a non-ccRCC patients receiving nephron-sparing treatments. Favorable discrimination was observed from the C-index (OS: 0.774, CSS: 0.801). The significant difference was observed between the low- and high-risk groups for both OS and CSS in KM curve analysis, implying that the nomograms exhibited outstanding ability for risk stratification. Calibration curves reflected the satisfactory accuracy of the OS and CSS nomograms. DCA proved the good clinical benefit and utility of the OS and CSS nomograms. The above results indicated the excellent performance of the nomograms, which would facilitate personalized treatment decisions and follow-up schedules.

To our knowledge, this is the first large-scale population-based study exploring the effects of LTD for cT1a nccRCC, developing and validating the prognostic nomograms for cT1a nccRCC patients receiving nephron-sparing treatments. However, the current study has some limitations. First, some bias was inevitable due to the retrospective design. Second, some important characteristics, such as comorbidities, tumor location, proximity of the tumor, and detailed treatment information were not available. Third, the principle of treatment has evolved toward more minimally invasive within the long-time span of the study, which could impact the development and application of PN and LTD treatment. Finally, the study is based on an American population cohort, the effects of LTD and PN on the treatment of cT1a nccRCC need to be further investigated in other countries. In the future, large-scale, well-designed prospective studies are needed to provide more high-quality evidence for the applications of LTD for nccRCC.

## Conclusions

This large-scale population-based comparative study demonstrated that OS of cT1a nccRCC patients receiving LTD was worse than patients receiving PN, whereas the CSS was similar between the two treatments. LTD could achieve comparable cancer-control effects as PN among cT1a nccRCC patients. Age, marital status, household income, prior tumor history, interval between diagnosis and treatment, treatments, and tumor size were identified as independent predictive factors for OS. Age, tumor size, and histological type were identified as independent predictive factors for CSS.Nomograms predicting OS and CSS were developed based on the above factors, which exhibited excellent performance in discrimination, risk stratification, accuracy, and clinical usefulness. This study could provide supporting evidence for applications of LTD in cT1a nccRCC patients, help to optimize individual treatment plans, and provide useful clinical prognostic predictive tools for cT1a nccRCC.

### Supplementary Information

Below is the link to the electronic supplementary material.

Supplementary file1 (DOCX 1285 KB)

## Data Availability

The SEER database released in May 2023 (https://seer.cancer.gov/data/) contained all reportable cancer cases from 17 registries (2000–2020) and provided complete information regarding demographics, cancer incidence, and survival.
