# Supplementary material for: The long-term outcomes of local tumor destruction versus partial nephrectomy for cT1a non-clear cell renal cell carcinoma and development of prognostic nomograms
Source: J Cancer Res Clin Oncol. 2024 Mar 12;150(3):122. doi: 10.1007/s00432-023-05571-8 (PMC10933168; doi:10.1007/s00432-023-05571-8)
Supplement: Supplementary file 1 — Supplementary file1 (DOCX 1285 KB) [file 432_2023_5571_MOESM1_ESM.docx]

The long-term outcomes of local tumor destruction versus partial nephrectomy for cT1a non-clear cell renal cell carcinoma and development of prognostic nomograms

**Journal name:** Journal of Cancer Research and Clinical Oncology

**Authors:**

Jianhui Qiu^1,2,3*^, Ruiyi Deng^1,2,3*^, Zihou Zhao^1,2,3^, Lin Cai^1,2,3^, Kan Gong^1,2,3^

**Author affiliation:**

1 Department of Urology, Peking University First Hospital, Beijing, China.

2 Institute of Urology, Peking University, Beijing, China.

3 National Urological Cancer Center, Beijing, China.

* These authors contributed equally to this work and should be considered co-first authors.

**Corresponding author:**

Kan Gong, MD., Prof.,

ORCID: 0000-0001-7195-677X

Department of Urology, Peking University First Hospital, Beijing, China. Institute of Urology, Peking University, Beijing, China. National Urological Cancer Center, Beijing, China.

No.8 Xishiku Street, Xicheng District, Beijing, P. R. China. 100034

Tel: (86)-010-83572075 Email: [kan.gong@bjmu.edu.cn](mailto:kan.gong@bjmu.edu.cn)

**Table S1.** Overall survival rates and cancer-specific survival rates of cT1a nccRCC patients before and after PSM.

| **Survival** | **Before PSM** | | **After PSM** | |
| --- | --- | --- | --- | --- |
|  | **PN** | **LTD** | **PN** | **LTD** |
| **Number of patients** | 2974 | 690 | 435 | 435 |
| **OS (%)** |  |  |  |  |
| 1 year | 98.4 | 96.8 | 96.5 | 96.9 |
| 3 years | 94.3 | 86.9 | 92.2 | 88.2 |
| 5 years | 89.0 | 76.0 | 85.3 | 80.2 |
| 10 years | 71.4 | 55.8 | 67.8 | 62.8 |
| **CSS (%)** |  |  |  |  |
| 1 year | 99.6 | 99.4 | 99.0 | 99.8 |
| 3 years | 98.8 | 97.3 | 98.3 | 97.9 |
| 5 years | 97.8 | 95.0 | 97.3 | 97.2 |
| 10 years | 93.6 | 91.2 | 95.1 | 94.7 |

**Table S2.** Overall survival rates and cancer-specific survival rates of cT1a nccRCC patients receiving different treatments.

| **Treatment** | **PN** | **Cryosurgery** | **Thermal ablation** | **Others** |
| --- | --- | --- | --- | --- |
| **Number of patients** | 2974 | 454 | 176 | 60 |
| **OS (%)** |  |  |  |  |
| 1 year | 98.4 | 97.2 | 97.0 | 93.2 |
| 3 years | 94.3 | 88.4 | 84.5 | 81.5 |
| 5 years | 89.0 | 76.5 | 76.1 | 72.2 |
| 10 years | 71.4 | 58.3 | 54.4 | 40.8 |
| **CSS (%)** |  |  |  |  |
| 1 year | 99.6 | 99.5 | 96.6 | 100 |
| 3years | 98.8 | 97.9 | 89.9 | 98.5 |
| 5years | 97.8 | 95.2 | 87.5 | 97.5 |
| 10 years | 93.6 | 91.6 | 87.5 | 91.1 |

**Table S3.** Baseline characteristics of cT1a nccRCC patients receiving nephron-sparing treatments in the overall cohort, the training cohort, and the validation cohort.

| **Characteristics** | | **Overall (n=3664)** | **Training cohort (n=2564)** | **Testing cohort (n=1100)** | **p-value** |
| --- | --- | --- | --- | --- | --- |
| **Age, n(%)** | <65 | 1640 (44.8) | 1141 (44.5) | 499 (45.4) | 0.86 |
|  | 65-85 | 1973 (53.8) | 1388 (54.1) | 585 (53.2) |  |
|  | >85 | 51 (1.4) | 35 (1.4) | 16 (1.5) |  |
| **Gender, n(%)** | Female | 895 (24.4) | 619 (24.1) | 276 (25.1) | 0.568 |
|  | Male | 2769 (75.6) | 1945 (75.9) | 824 (74.9) |  |
| **Race, n(%)** | White | 2793 (76.2) | 1942 (75.7) | 851 (77.4) | 0.425 |
|  | Black | 754 (20.6) | 542 (21.1) | 212 (19.3) |  |
|  | Others | 117 (3.2) | 80 (3.1) | 37 (3.4) |  |
| **Marital status, n(%)** | None/unknown | 1254 (34.2) | 877 (34.2) | 377 (34.3) | 0.998 |
|  | Yes | 2410 (65.8) | 1687 (65.8) | 723 (65.7) |  |
| **Median household income, n(%)** | $ (0-75,000) | 2036 (55.6) | 1415 (55.2) | 621 (56.5) | 0.502 |
|  | $ (75,000+) | 1628 (44.4) | 1149 (44.8) | 479 (43.5) |  |
| **Rural/urban population density, n(%)** | Counties in metropolitan areas ge 1 million pop | 2360 (64.4) | 1665 (64.9) | 695 (63.2) | 0.585 |
|  | Counties in metropolitan areas of 0 to 1 million pop | 973 (26.6) | 668 (26.1) | 305 (27.7) |  |
|  | Nonmetropolitan counties | 327 (8.9) | 229 (8.9) | 98 (8.9) |  |
|  | Unknown | 4 (0.1) | 2 (0.1) | 2 (0.2) |  |
| **Laterality, n(%)** | Left | 1818 (49.6) | 1282 (50.0) | 536 (48.7) | 0.503 |
|  | Right | 1846 (50.4) | 1282 (50.0) | 564 (51.3) |  |
| **Prior tumor number, n(%)** | 0 | 1405 (38.3) | 987 (38.5) | 418 (38.0) | 0.197 |
|  | 1 | 1838 (50.2) | 1268 (49.5) | 570 (51.8) |  |
|  | >2 | 421 (11.5) | 309 (12.1) | 112 (10.2) |  |
| **Prior tumor behavior, n(%)** | No prior tumor | 1405 (38.3) | 987 (38.5) | 418 (38.0) | 0.973 |
|  | Benign prior tumor | 56 (1.5) | 38 (1.5) | 18 (1.6) |  |
|  | Borderline /in situ prior tumor | 139 (3.8) | 96 (3.7) | 43 (3.9) |  |
|  | Malignant prior tumor | 2064 (56.3) | 1443 (56.3) | 621 (56.5) |  |
| **Prior tumor surgery treatment, n(%)** | No prior tumor surgery treatment | 1913 (52.2) | 1357 (52.9) | 556 (50.5) | 0.233 |
|  | Prior tumor surgery treatment | 1645 (44.9) | 1129 (44.0) | 516 (46.9) |  |
|  | Unknown | 106 (2.9) | 78 (3.0) | 28 (2.5) |  |
| **Prior tumor radiotherapy, n(%)** | No prior tumor | 1405 (38.3) | 987 (38.5) | 418 (38.0) | 0.819 |
|  | None/Unknown | 1792 (48.9) | 1246 (48.6) | 546 (49.6) |  |
|  | Yes | 467 (12.7) | 331 (12.9) | 136 (12.4) |  |
| **Prior tumor chemotherapy, n(%)** | No prior tumor | 1405 (38.3) | 987 (38.5) | 418 (38.0) | 0.54 |
|  | No prior tumor chemotherapy/unknown | 1896 (51.7) | 1315 (51.3) | 581 (52.8) |  |
|  | Yes | 363 (9.9) | 262 (10.2) | 101 (9.2) |  |
| **Interval between diagnosis and treatment (mean [SD])** | | 1.26 [2.22] | 1.21 [2.13] | 1.39 [2.40] | 0.738 |
| **Tumor size, n(%)** | <1 cm | 131 (3.6) | 97 (3.8) | 34 (3.1) | 0.338 |
|  | 1-2 cm | 1160 (31.7) | 801 (31.2) | 359 (32.6) |  |
|  | 2-3 cm | 1430 (39.0) | 1013 (39.5) | 417 (37.9) |  |
|  | 3-4 cm | 943 (25.7) | 653 (25.5) | 289 (26.3) |  |
| **Grade, n(%)** | Grade I | 425 (11.6) | 295 (11.5) | 130 (11.8) | 0.978 |
|  | Grade II | 1491 (40.7) | 1047 (40.8) | 444 (40.4) |  |
|  | Grade III | 610 (16.6) | 425 (16.6) | 185 (16.8) |  |
|  | Grade IV | 35 (1.0) | 23 (0.9) | 12 (1.1) |  |
|  | Unknown/unapplicable | 1103 (30.1) | 774 (30.2) | 329 (29.9) |  |
| **Histology, n(%)** | pRCC | 2786 (76.0) | 1957 (76.3) | 829 (75.4) | 0.959 |
|  | chRCC | 756 (20.6) | 521 (20.3) | 235 (21.4) |  |
|  | Cyst-associated RCC | 95 (2.6) | 67 (2.6) | 28 (2.5) |  |
|  | Sarcomatoid RCC | 19 (0.5) | 13 (0.5) | 6 (0.5) |  |
|  | Collecting duct RCC | 8 (0.2) | 6 (0.2) | 2 (0.2) |  |


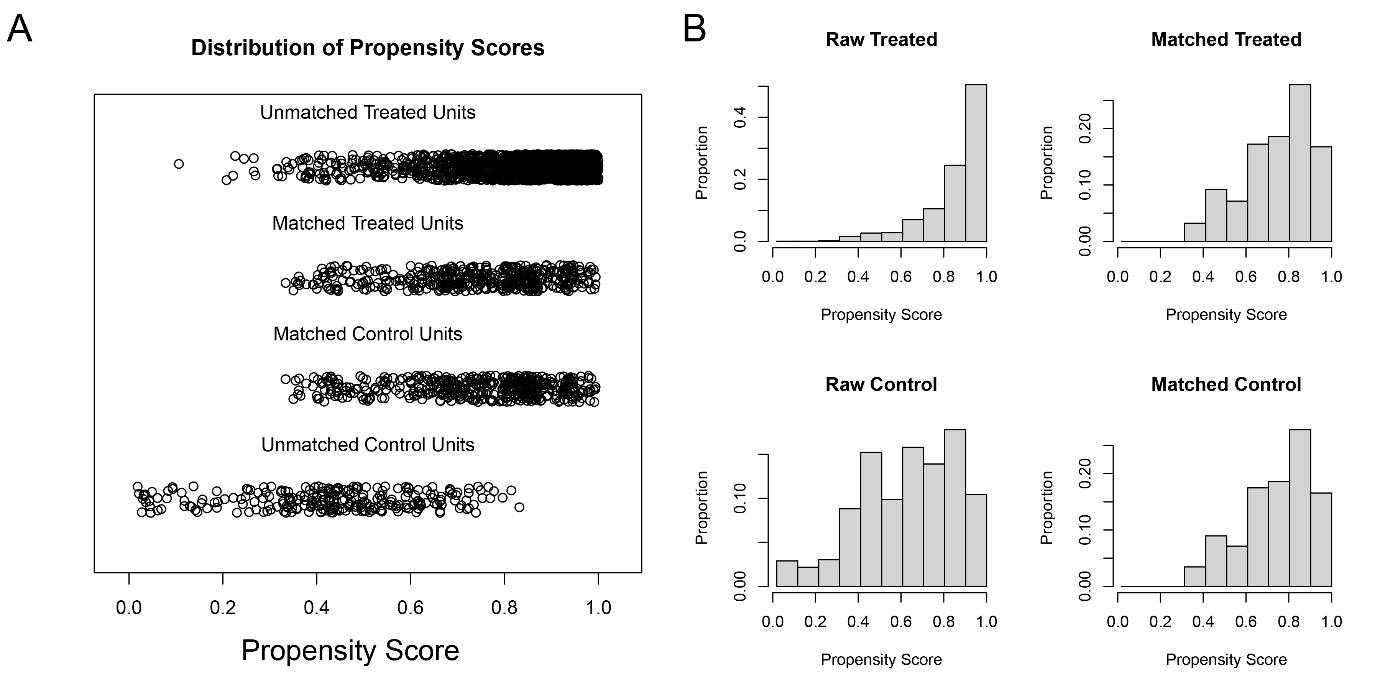


**Fig S1.** (A) Distribution of propensity score. (B) Histogram of propensity score.


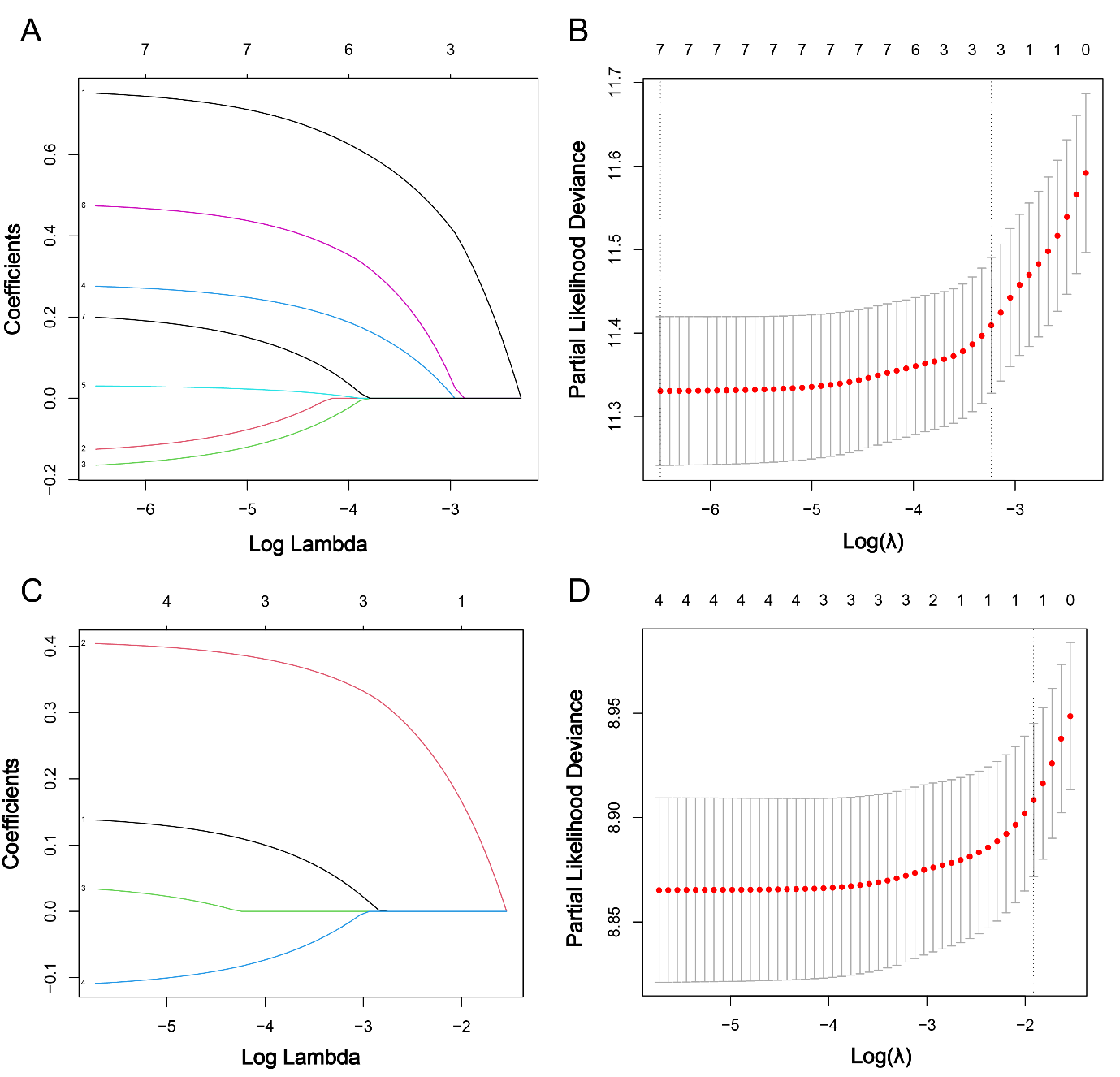


**Fig S2.** (A) LASSO coefficient profiles of 7 variables for OS; (B) LASSO analysis identified 7 variables for OS; (C) LASSO coefficient profiles of 4 variables for CSS; (D) LASSO analysis identified 4 variables for CSS.


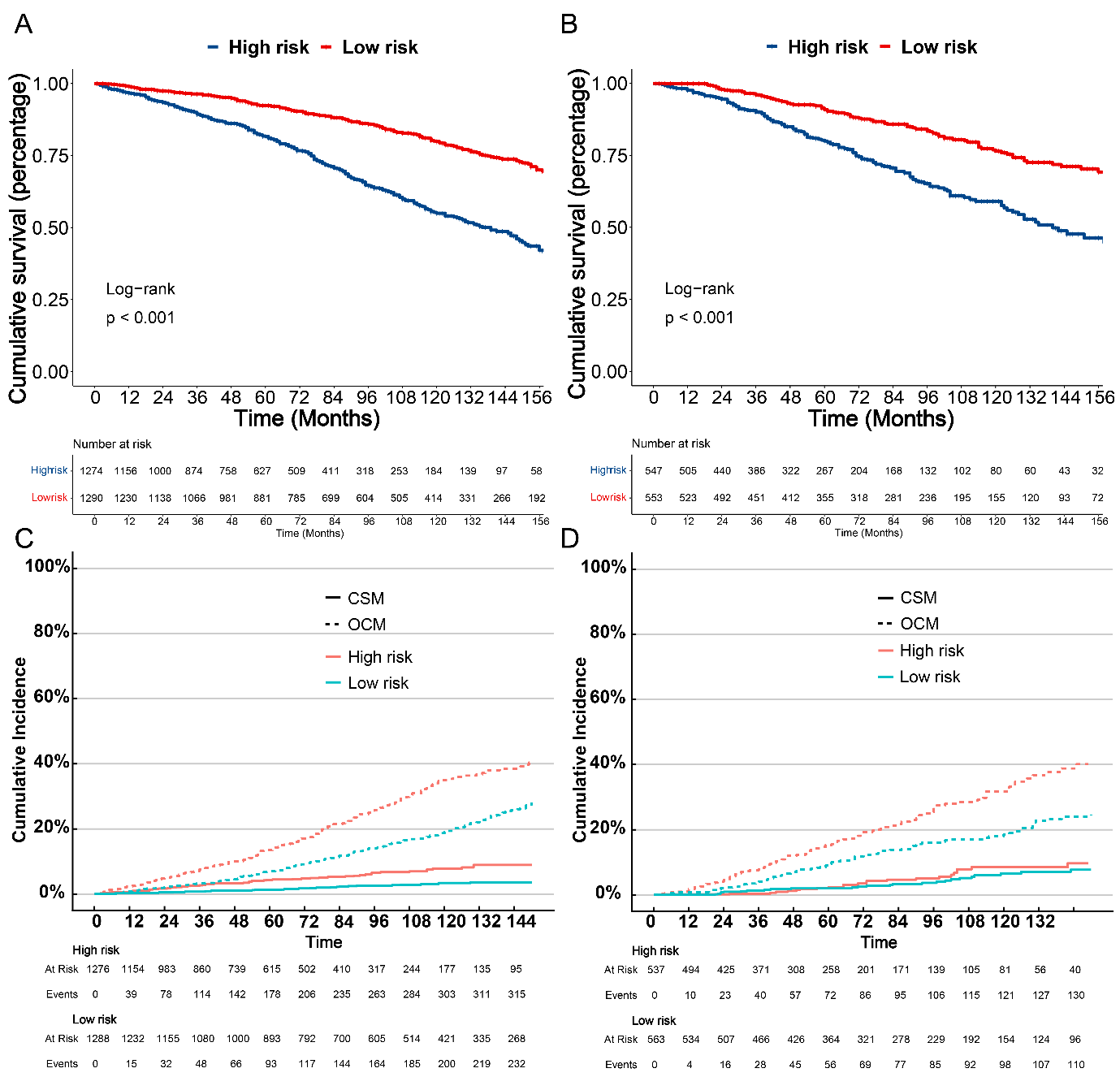


**Fig S3.** Kaplan‒Meier curves of OS for cT1a nccRCC patients receiving nephron-sparing treatments in the training set (A) and the validation set (B) stratified by the risk stratification system. Cumulative incidence plots for cT1a nccRCC patients receiving nephron-sparing treatments in the training set (C) and the validation set (D) stratified by the risk stratification system.


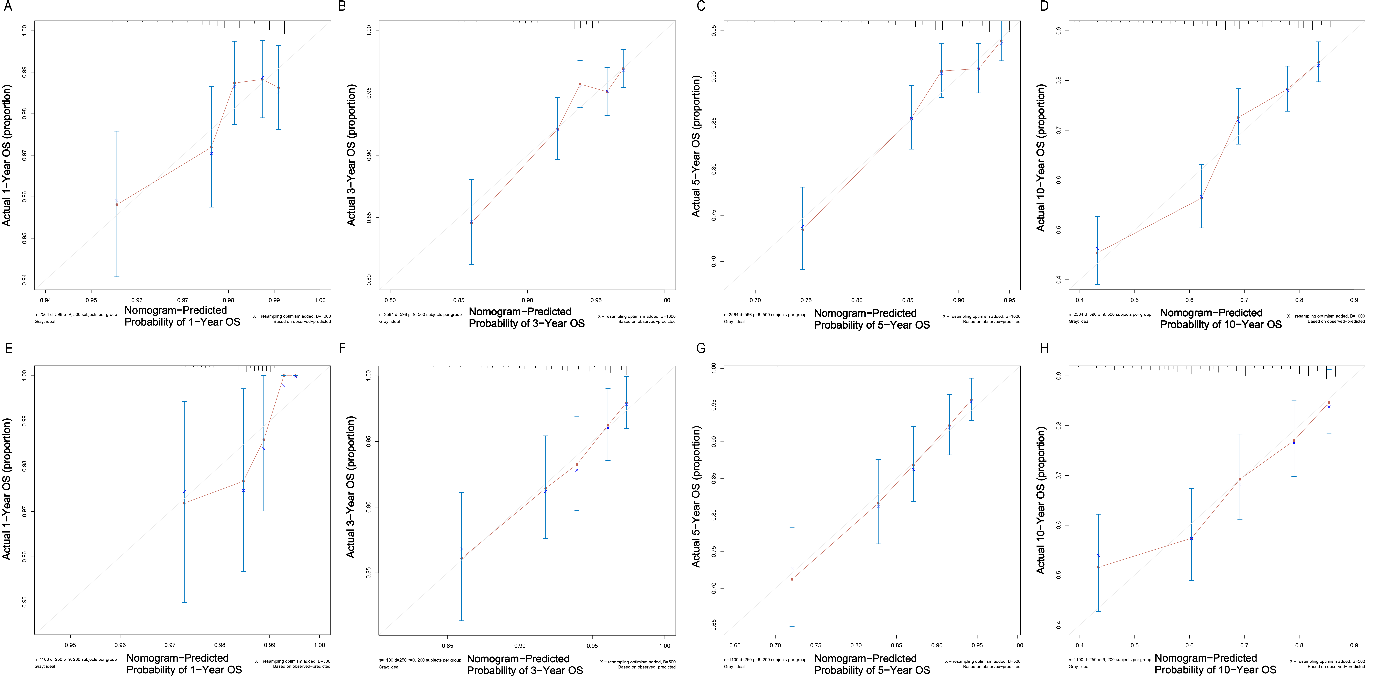


**Fig S4.** The calibration curves of the nomogram evaluating the 1-year, 3-year, 5-year, and 10-year OS of cT1a nccRCC patients receiving nephron-sparing treatments in the training set (A-D) and the validation set (E-H).


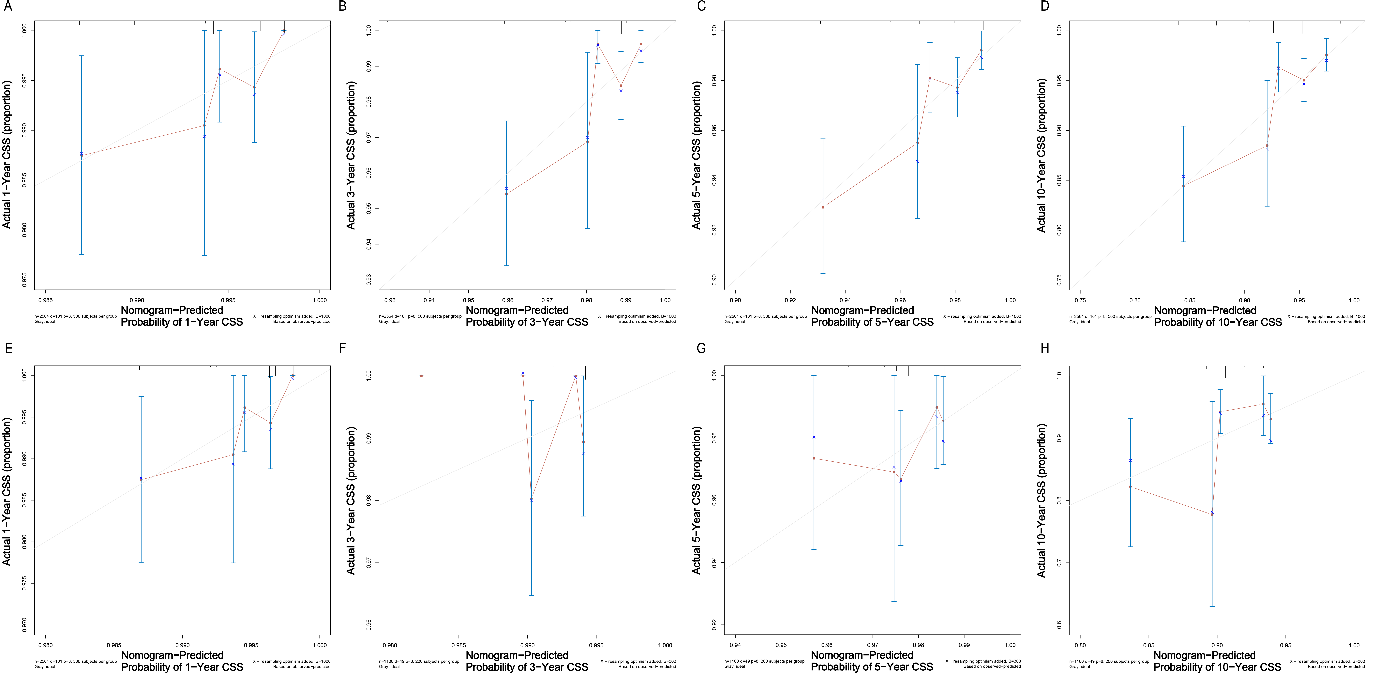


**Fig S5.** The calibration curves of the nomogram evaluating the 1-year, 3-year, 5-year, and 10-year CSS of cT1a nccRCC patients receiving nephron-sparing treatments in the training set (A-D) and the validation set (E-H).


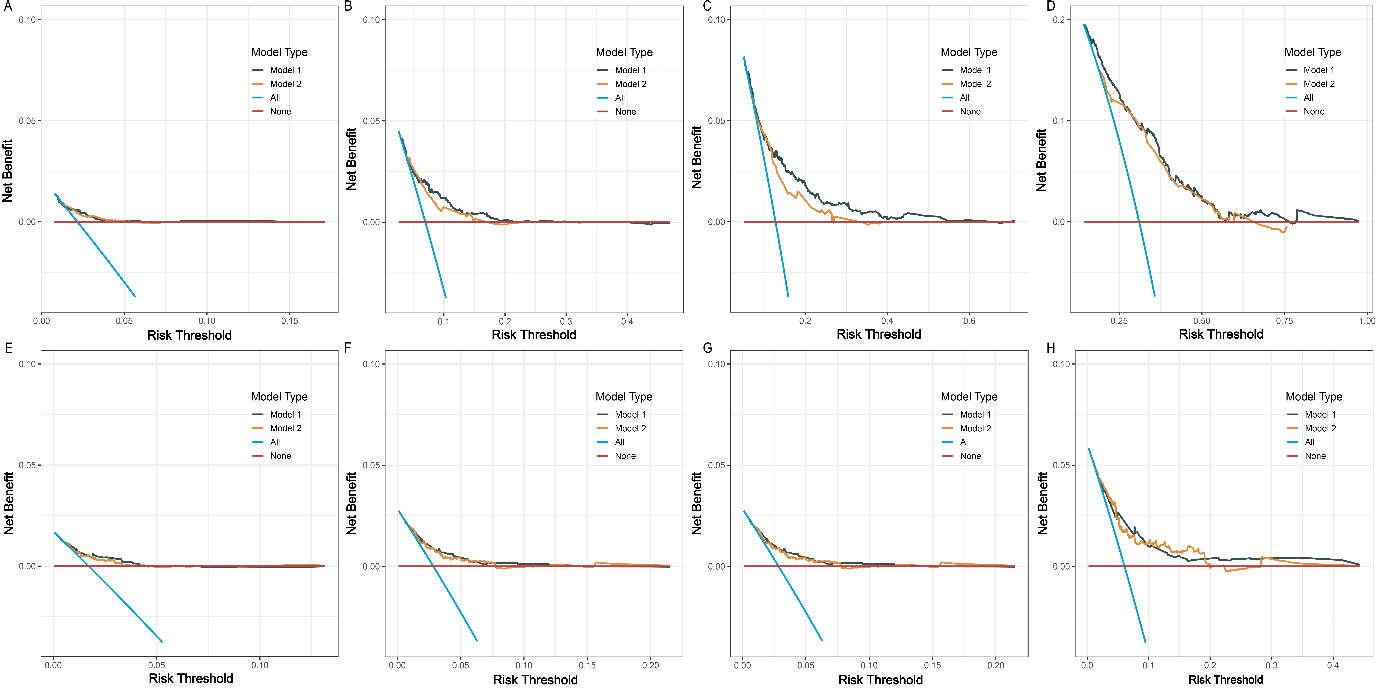


**Fig S6.** Decision curve analysis of the nomogram evaluating the 1-year, 3-year, 5-year, and 10-year OS (A-D) and CSS (E-H) of cT1a nccRCC patients receiving nephron-sparing treatments. Model 1, predictive model of this study. Model 2, previous predictive model.
